# Wellbore Skin: Why Its Presence and Properties Are So Difficult to Predict

**DOI:** 10.1111/gwat.13498

**Published:** 2025-07-10

**Authors:** Georg J. Houben, Matthias Halisch, Reiner Dohrmann, Axel Lamparter, Kristian Ufer, Christin Damian, Daniel Boz

**Affiliations:** ^1^ Federal Institute for Geosciences and Natural Resources, BGR Stilleweg 2 30655 Hannover Germany; ^2^ LIAG Institute for Applied Geophysics Hannover Germany; ^3^ State Authority for Mining, Energy and Geology of Lower Saxony (LBEG) Hannover Germany; ^4^ Lausitz Energie Bergbau AG (LEAG) Boxberg Germany

## Abstract

The presence of positive wellbore skin, that is, deposits of fine‐grained particles from the drilling fluid on the borehole wall, significantly affects the efficiency of water wells. Previous studies of skin samples have shown a significant variability in typology, thickness, and composition but were largely unable to explain the differences. In order to overcome this problem, we therefore (1) significantly expanded the sample data base by investigating skin samples from nine wells with very similar geological and technical conditions and (2) investigated the evolution of the density of drilling fluids during the drilling. The former is done in order to evaluate differences in skin thickness and composition, and the latter to study the differential mobilization of particles. Incohesive and poorly sorted layers form the source of the particles, while the thickest accumulation of particles occurs in highly permeable layers, where the highest exfiltration rates initially occur. For well drillers, we recommend continuous monitoring of drilling fluid density to obtain a measure of the presence of particle‐providing layers and the probability of wellbore skin formation.

## Introduction

During the drilling of a well, particles from incohesive and poorly sorted sediment layers can become mobilized into the drilling fluid and re‐deposited around the borehole wall. This so‐called positive wellbore skin decreases the hydraulic conductivity of the near field of the borehole (Hurst 1953; van Everdingen 1953; Abboud and Corapcioglu 1993; Loeber et al. 1996). Since the presence of such a barrier diminishes drilling fluid losses and thus helps prevent borehole collapse, fine‐grained sealing material is often intentionally added to the drilling fluid, for example, in the form of swellable clay (bentonite) or carboxymethylcellulose (CMC). In the later operational phase, the presence of wellbore skin is undesirable, since its low permeability significantly hinders water flow into the well. This results in significant additional head losses, which require extra energy to overcome and may negatively influence the interpretation of pumping tests (Houben 2015). Considering the large amounts of energy spent on operating the tens of millions of wells in operation worldwide, understanding and predicting the formation of wellbore skin formation is thus highly desirable.

**Figure 1:**
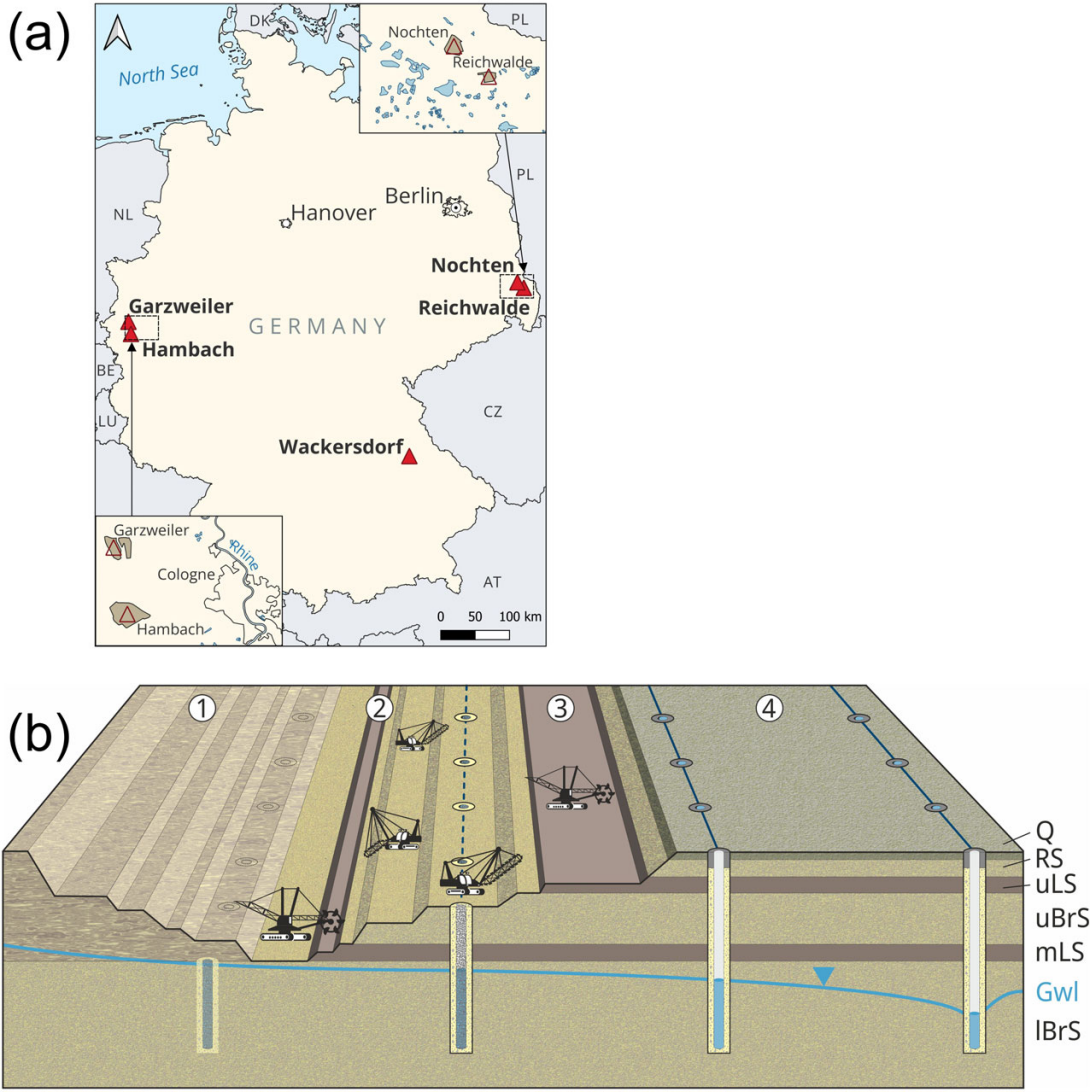
(a) location map of the lignite mines Reichwalde and Nochten and other sites mentioned in the text, (b) schematic cross‐section of a Lusatian lignite mine (not to scale). 1 = mine waste backfill, 2 = main seam, 3 = upper seam, 4 = foreland with dewatering well rows, Q = Quaternary, RS = Raunoer Series, uLS = upper Lusatian lignite seam, uBrS = upper Briesker Series (aquifer), mLS = main Lusatian lignite seam, lBrS = lower Briesker Series (aquifer), Gwl = groundwater level.

However, a general problem of research on wellbore skin is the scarcity of actual samples from the borehole wall, which are difficult and expensive to obtain. To overcome this, Houben et al. (2016, 2024) analyzed wellbore skin samples taken from dewatering wells of open‐pit lignite mines in several regions of Germany (Figure 1). These wells had been exposed by the advancing mine itself after several years of operation. Remarkable variations in typology, thickness, composition, grain size distribution, and hydraulic conductivities were found, but no satisfactory explanations for the observed differences in thickness.

Therefore, we expand the data base on wellbore skin significantly by studying wellbore skin material from nine technically identical dewatering wells from the Reichwalde lignite mine, all drilled into the same formation at roughly the same time. In order to study the mobilization of particles, we incorporated the analysis of the drilling fluid density of six wells from the neighboring Nochten mine (Figure 1). The main aim was to find an explanation for the observed variability in wellbore skin composition and especially thickness, focusing on the role of source and sink layers for particles. The findings will help well operators to better monitor, predict, and counteract the formation of wellbore skin.

## Research Method

The main study sites, the mines Reichwalde and Nochten, are located in the Lusatian lignite mining district, roughly 140 km southeast of Berlin, Germany (Figure 1). The mines are operated by the Lausitz Energie Bergbau AG (LEAG). In both, two Miocene seams are mined: the thinner upper First Lusatian seam (≈3 m thickness) and the lower, main Second Lusatian seam (up to 15 m; Nowel et al. 1994). The former is overlain by the Cenozoic Raunoer Series, consisting of terrestrial fluvial deposits and a Quaternary cover.

For mine dewatering, several rows of abstraction wells are drilled parallel to the advancing mine front (Figure 1b). The distance between the individual wells is about 100 m. All encountered aquifers are dewatered simultaneously through one continuous well screen. The advancing mine front wells excavate the dewatering wells after a few years (here: five), allowing inspection and sampling from the outside.

Here, samples of the borehole wall from nine dewatering wells of well row FR47 of the Reichwalde mine were investigated (Figure 1, Table 1). All wells had been drilled by the same company within 3 months (November 2017 to January 2018) and are identical regarding drilling technique (airlift), drill rig, and well completion. The first five meters were drilled with a diameter of 1000 mm, followed by a diameter of 750 mm until the final depth. The screen consists of a pre‐glued gravel pack tube of 350 mm diameter and 30 mm thickness, surrounded by a gravel pack (grain size range of 0.71–3.15 mm). No drilling fluid additives were used; thus, any wellbore skin encountered is entirely due to particle uptake from the perforated formations. Unlike most drinking water wells, the wells were not developed after drilling due to their expected short life span. Here, we study three groups of wells within the well row, the first consisting of wells Br 24, 25, and 26, the second of wells Br 33, Br 34, 34A, and 35, and the third of wells Br 40 and 41. One drill hole, Br. 34, had to be abandoned at a depth of 60 m and was replaced by a new one, Br 34A, drilled only 6 m away, which reached the intended final depth. The pair Br 34 and Br 34A allows the comparison of the influence of penetrating the main lignite seam, since the former did not reach it. Sampling took place at the first mine floor level (≈130 m asl), corresponding to depths from the original surface of around 6–19 m. The sediments at this depth are of Pleistocene age.

**Table 1:** General Information on the Wells from the Reichwalde (RW) Lignite Mine, after Data by LEAG

Code	Construction Year	Ground Elevation (m asl)	Bottom Elevation (m asl)	Final Depth (m)	Sample Depth (m asl)
FR47/24	2018	147.5	43.5	104	128.7
FR47/25	2017	136.9	44.9	92	129.4
FR47/26	2017	141.5	45.5	96	129.8
FR47/33	2017	143.8	47.8	96	131.4
FR47/34	2017	139.8	79.8	60	131.5
FR47/34A	2017	139.8	48.8	91	131.5
FR47/35	2017	137.9	48.9	89	131.8
FR47/40	2017	143.8	50.8	93	132.0
FR47/41	2017	142.1	52.1	90	131.7

The saturated hydraulic conductivity was measured with a constant head laboratory permeameter (Eijkelkamp, The Netherlands), using tap water at 20 °C. Measured values were corrected to a water temperature of 10 °C. The horizontally oriented core plugs of 80 mm diameter and 50 mm height were directly taken in the field. The wellbore skin is too thin and fragile for an individual permeability analysis. Instead, core plugs of the borehole wall, comprising a sequence of all three materials (gravel, skin, and aquifer) were taken and analyzed. With the individually measured hydraulic conductivities of the gravel pack, the aquifer, and the sequence plug, the hydraulic conductivity of the skin layer could be recalculated (Domenico and Schwartz 1996; Houben et al. 2024). The skin layer thickness was measured after opening the core.

Unconsolidated samples were stabilized using the two‐component epoxy resin Araldite® 2020 (XW 396/XW 397), for example, for later thin section and computed tomography analysis. The resin has a density of 1.1 kg/m^3^ and an initial dynamic viscosity of 150 mPa·s. Samples were pre‐heated to 40 °C to improve resin penetration.

Optical analysis on resin‐stabilized thin sections was done using a Zeiss Axioplan polarizing microscope (transmitted light) and the Zeiss Axiovision Software. For higher resolutions, a Zeiss Sigma 300VP Scanning Electron Microscope (SEM) was used (air dried samples). The element spectra were obtained with an attached Bruker XFlash 6|60.

Mineral phases were identified using X‐ray diffraction (XRD), using a PANalytical X'Pert PRO MPD Θ‐Θ diffractometer, with Co‐Kα radiation at 40 kV and 40 mA and a variable divergence slit (20 mm irradiated length), combined with primary and secondary soller, diffracted beam monochromator, point detector, and a sample changer (diameter 28 mm). All samples were scanned from 3° to 80° 2Θ, utilizing a step size of 0.03° 2Θ and a total measuring time of 2 h. Quantitative data were obtained by Rietveld refinement of the XRD data, using the software package Profex/BGMN (Bergmann et al. 1998; Döbelin and Kleeberg 2015).

For granulometric analysis, the fraction >63 μm was separated by manual sieving and the grain size of 10 g of dried and disaggregated material was analyzed optically with a CAMSIZER P4 (Retsch, Germany). The grain size distribution of the fraction <63 μm, if present, was determined using X‐ray granulometry (XRG) with a SediGraph 5100™ (Micrometrics, USA). All samples were therefore dispersed in a 0.01 N Na_2_P_2_O_7_·10 H_2_O solution, freeze‐dried, and re‐suspended, the latter step including a two‐step ultrasonic treatment (each 2 min, 20 kHz).

The particles that make up the wellbore skin must come from the drilling fluid. Therefore, the drilling fluid of six boreholes was sampled during drilling. Samples were taken from wells of the Nochten mine, wells Br. 5 and 14 of well row FR 152 and Br. 7, 8, 9, and 12 of well row FR 156. Samples were taken at intervals of 10 m of depth, for the first two plus one additional sample at the top of the main coal seam. They were taken from the drilling fluid circle after passing the settlement pit, immediately before reinjection. Remainders of the sand fraction were removed by manual sieving (<63 μm) in the field. The density of the drilling fluid was determined at full suspension and after 30 min of settlement, using a DMA 35 density meter by Anton Paar (Graz, Austria), which uses the oscillating U‐tube method. Bi‐distilled water was used as standard. The particle load was obtained from the measured bulk density by subtracting the density of pure groundwater. All measured densities were converted to values for 20 °C. The grain size distribution of the suspended matter for the first two samples was analyzed with XRG as described above.

For the 3D imaging, a high‐resolution X‐ray computed tomography (μ‐CT) system nanotom M 180 (Baker Hughes Waygate), operated by LIAG (Hanover, Germany) was used. It is equipped with a high‐flux water‐cooled nanofocus X‐ray tube (180 kV, 20 W) and a large, water‐cooled detector (9 MPx, 12 ms acquisition time) with a high contrast‐to‐noise ratio (typically better than 10,000:1). More details about the scanning procedure are found in Halisch et al. (2016). The digital image processing was performed using Avizo (2019) software toolbox. For this study, three typical samples were selected (Br 24, Br 26, Br 41). They were cut from the resin‐bound core slices described above. The voxel resolution was 7.0 μm. The individual image slices were combined into animations (see Supporting Information).

## Results

### Density of Drilling Fluid and Grain Size Distribution of Suspended Matter

Particles suspended in the drilling fluid are the source material for skin formation. Its particle load can be estimated from its density. Measurements of the density of the fully re‐suspended drilling fluid samples showed significant differences between the wells, even if coming from the same well row, despite all wells being very similar in terms of well construction and completion.

**Figure 2:**
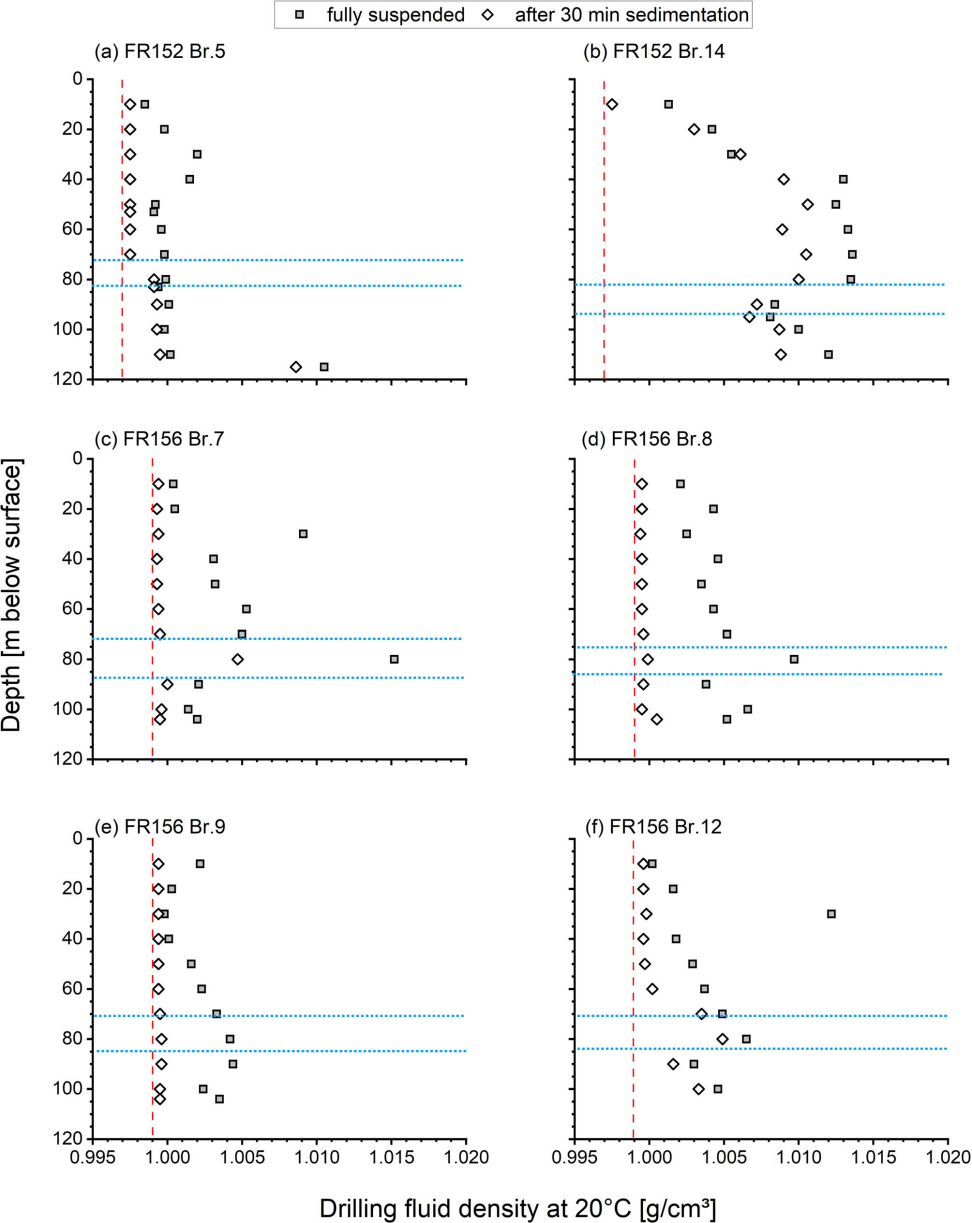
Depth‐specific drilling fluid density of wells Br. 5 and Br. 14 (a, b) from well row FR 152 and wells Br. 7, 8, 9, 12 from well row FR156, both in Nochten. The red lines indicate the density of groundwater; the blue lines indicate the position of the main lignite seam.

The drilling fluid in well Br 5 mobilized only a small particle load, evidenced by the low densities. The relatively high density of the lowermost sample is probably related to settlement processes in the borehole. On the other hand, the drilling fluid of well Br 14 attained significantly higher particle loads (Figure 2). They increase almost linearly down to a depth of ca. 40 m, then remain constant until reaching the main lignite seam, when densities decrease somewhat, followed again by an increase. Wells Br 9 and 12 also show an almost linear increase of densities with depth, while well Br 7 exposes some jumps and Br 8 an almost constant density throughout. With the exception of well Br 5, the density decreases after reaching the bottom of the main seam.

Another difference lies in the particle sedimentation behavior. Therefore, the fully re‐suspended suspensions were allowed to settle for 30 min, and the densities of the supernatant fluid were measured again (Figure 2). For Br 5, samples above the main lignite seam lost most of their particle load over this period of time, indicating a predominantly silty composition, with little clay present (Figure 2). Below the coal seam, the suspended material seems to be finer and less prone to gravitational settling. The material suspended in the drilling fluid of well Br 14 is generally less susceptible to sedimentation. With the exception of the uppermost sample, only 10–25% of the particle load settles out after 30 min, indicating a very fine, clay dominated grain spectrum (Figure 2). Wells Br 7, 8, and 9 also show a fast settlement rate, while well Br 12 shows a higher percentage of clay particles, at least at deeper depths.

**Figure 3:**
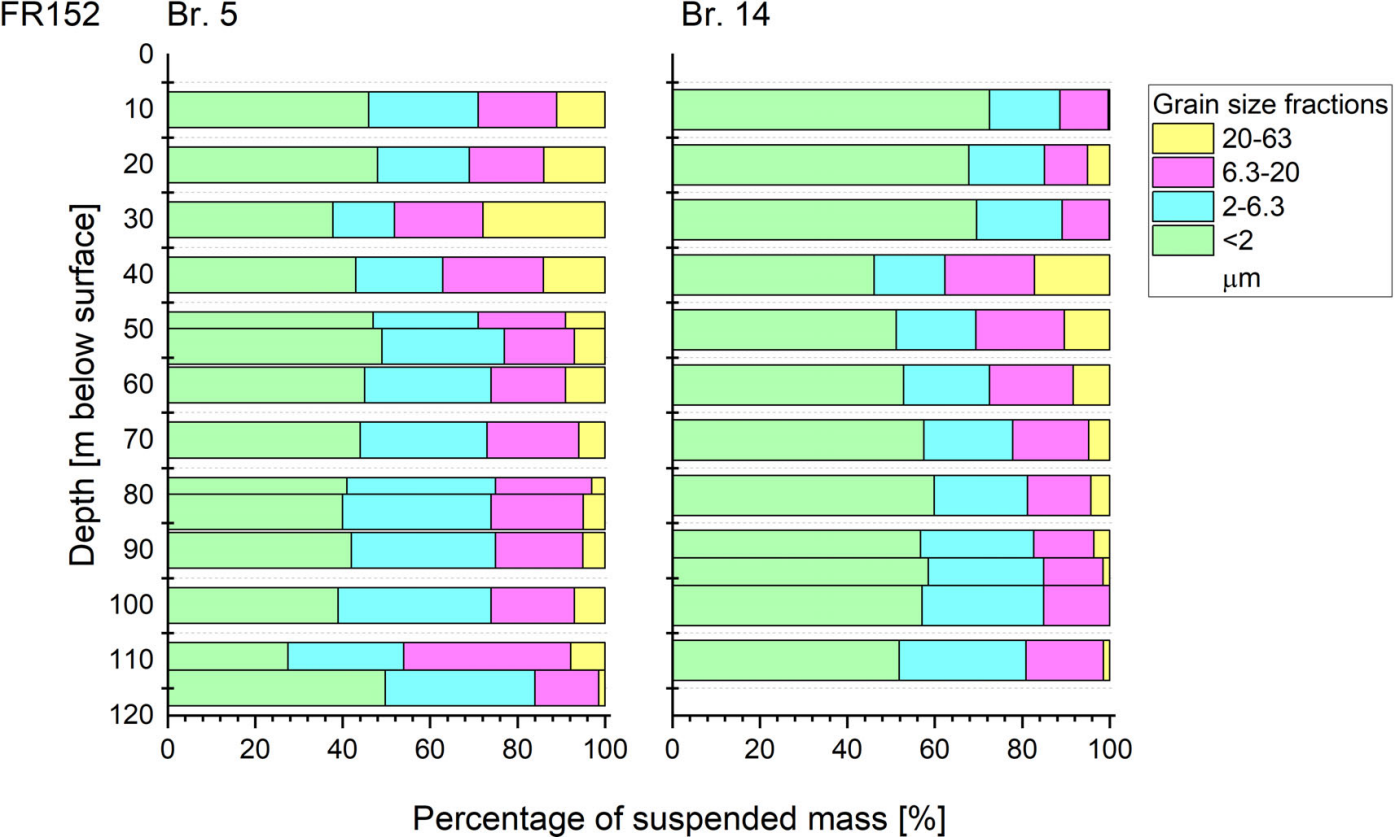
Grain size fractions as percentages of mass suspended in the drilling fluid of wells Br. 5 and Br. 14 from well row FR 152, Nochten. 20–63 μm = coarse silt, 6.3–20 μm = medium silt, 2–6.3 μm = fine silt, <2 μm = clay.

The relative proportions of silt and clay fractions in the suspended particle load of the drilling fluid were measured using the sedigraph for two samples (Figure 3). In order to relate these values to the particle load, the bulk density of water (0.9973 g/cm^3^ at 20 °C) was deducted. The results show that the suspended material from Br 14 generally has a higher relative proportion of clay compared to Br 5 (Figure 3). This is in good accordance with the sedimentation behavior described above. The jump in density at around 40 m depth is mainly caused by an increase in the silt fractions, indicating an origin from the poorly sorted silt layer described below.

The reason for the different drilling fluid densities and thus particle loads has to be sought in the geological heterogeneity. Significant geological heterogeneity can already occur on a small spatial scale. Here, the potential sources for mobilized particles comprise clay layers, poorly sorted silty to sandy sediments, and the lignite seams. According to the drill logs, the upper lignite seam at Br 5 is overlain by a clay layer of 1 m thickness (1–2 m depth). Due to its shallow depth, it might have been above the water table in the active borehole and thus unable to provide particles. A clay layer of 3 m thickness was found in Br 14 at a depth of 5–8 m, which is probably the source of the initial increase of particle load. However, not all clay layers seem to be relevant particle providers. The geological log of well Br 14 indicates the presence of a clay layer of 3 m thickness at around 62–65 m depth. This layer, however, does not show up in the particle load distribution in Figure 2. Thus, not all clay layers are necessarily significant particle sources. Clay can be quite cohesive, preventing its erosion from the borehole wall. The most significant jump in the density of the drilling fluid at Br 14 occurs at a depth of around 40 m, which coincides with a silt layer of 6 m thickness (32–38 m). At well Br 5, a 6 m silty fine sand layer is found at the same position. The difference between a silt and a silty fine sand is thus apparently sufficient to significantly influence the mobilized particle load. Whether such small differences can be distinguished properly in the field is up to debate.

The role of the lignite seams as particle sources requires separate attention. Previous studies by Houben et al. (2016, 2024) and the analyses of the Reichwalde wells discussed below clearly show that mobilized lignite material forms a significant part (by volume) of the wellbore skin. Although the drilling fluid for both wells attains a dark color after reaching the main lignite seam, the suspended particle load remains the same for Br 5 or even decreases somewhat for Br 14 (Figure 2). The contribution of the lignite seam to the mass of the suspended particle load is thus smaller than its volumetric contribution. The much lower density of lignite material (650–850 kg/m^3^) compared to silicates (e.g., quartz at 2650 kg/m^3^) is the most probable explanation.

### Typology and Thickness of Wellbore Skin

**Figure 4:**
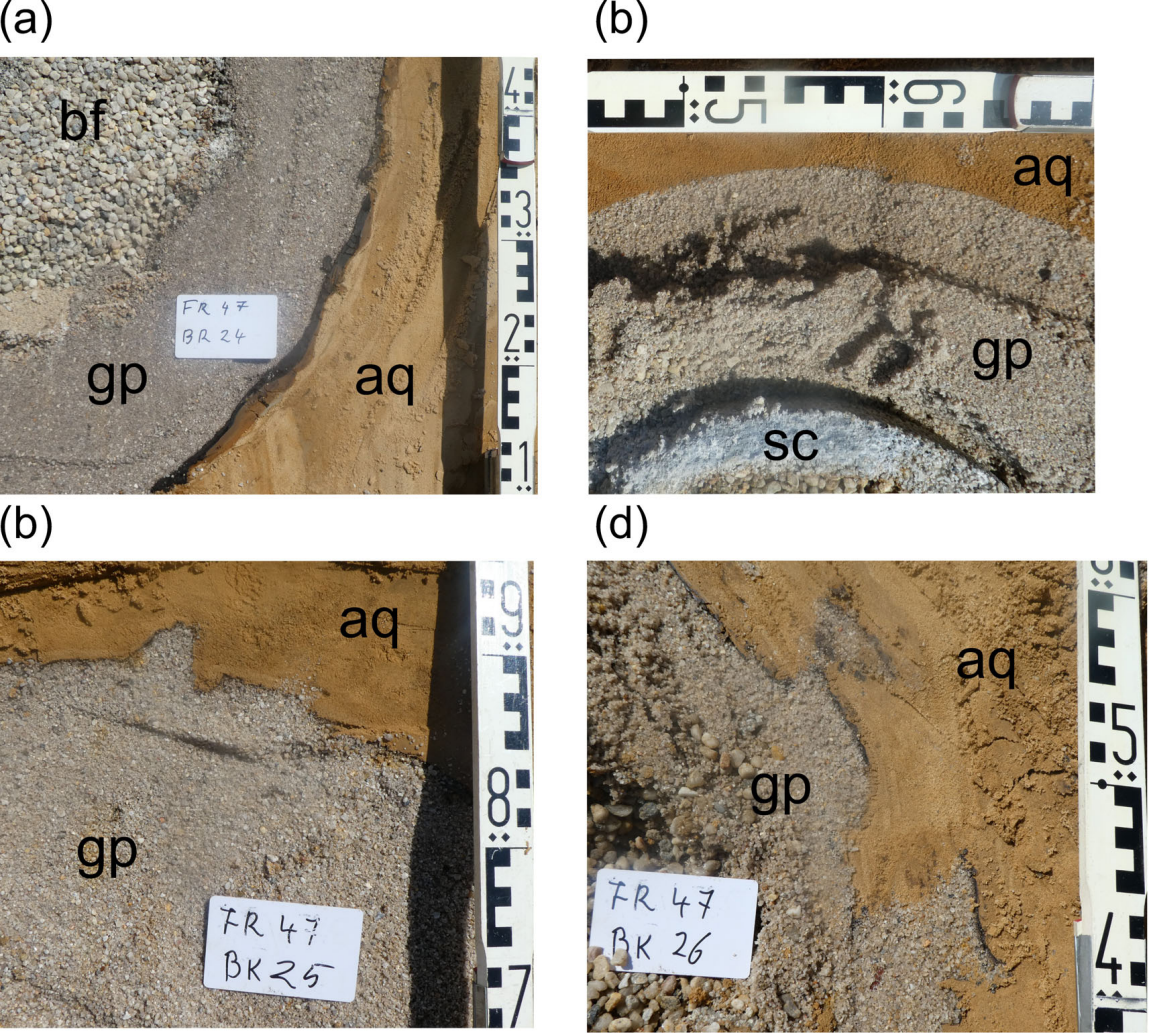
View of typical features on borehole walls of the Reichwalde wells (field scale): (a) Br 24: wellbore skin layer of variable thickness, locally extremely thick, with layering (inner side dark, outer side brown), (b) Br 26: very thin to absent wellbore skin, (c) Br 25: borehole wall discontinuity (partially plugged by skin material), (d) Br 26: borehole wall discontinuity (partially plugged by skin material). bf = backfill (well interior), sc = screen tube, gp = gravel pack, aq = aquifer. Wellbore skin, if present, is found between gravel pack and aquifer.

Despite their similarity in design and being installed in the same geological formation, the nine Reichwalde wells showed a marked variability in well bore skin thickness. In some wells, it was very thin (<1 mm, e.g., Br 26 [Figure 4b], Br 34, Br 40, Br 41), while others showed the typical thicknesses of 1–2 mm (e.g., Br. 25, Br 34A, and Br 35) observed in previous studies (Houben et al. 2016, 2024). In some wells, the thickness varied significantly along the borehole wall (e.g., Br 25, Br 33, and Br 40). Br 24 showed the highest skin thicknesses observed so far, ranging from 10 to 25 mm, an order of magnitude higher than previously recorded (Figure 4a). It also showed a distinct zonation, with an inner (toward the gravel pack) dark layer and an outer (toward the borehole wall) brownish layer.

Houben et al. (2024) had first documented the presence of small discontinuities in the wellbore skin deposits (wormholes), which can act as preferential flow paths. They can form via mechanical stresses acting upon the borehole wall, for example, during the backfilling of the gravel pack or through later localized inflow. Some of the Reichwalde wells studied here showed similar effects (Figure 4c and 4d). However, some of the discontinuities found here had been plugged with fine‐grained material.

### Microscopic and Electron Microscopic Analysis

**Figure 5:**
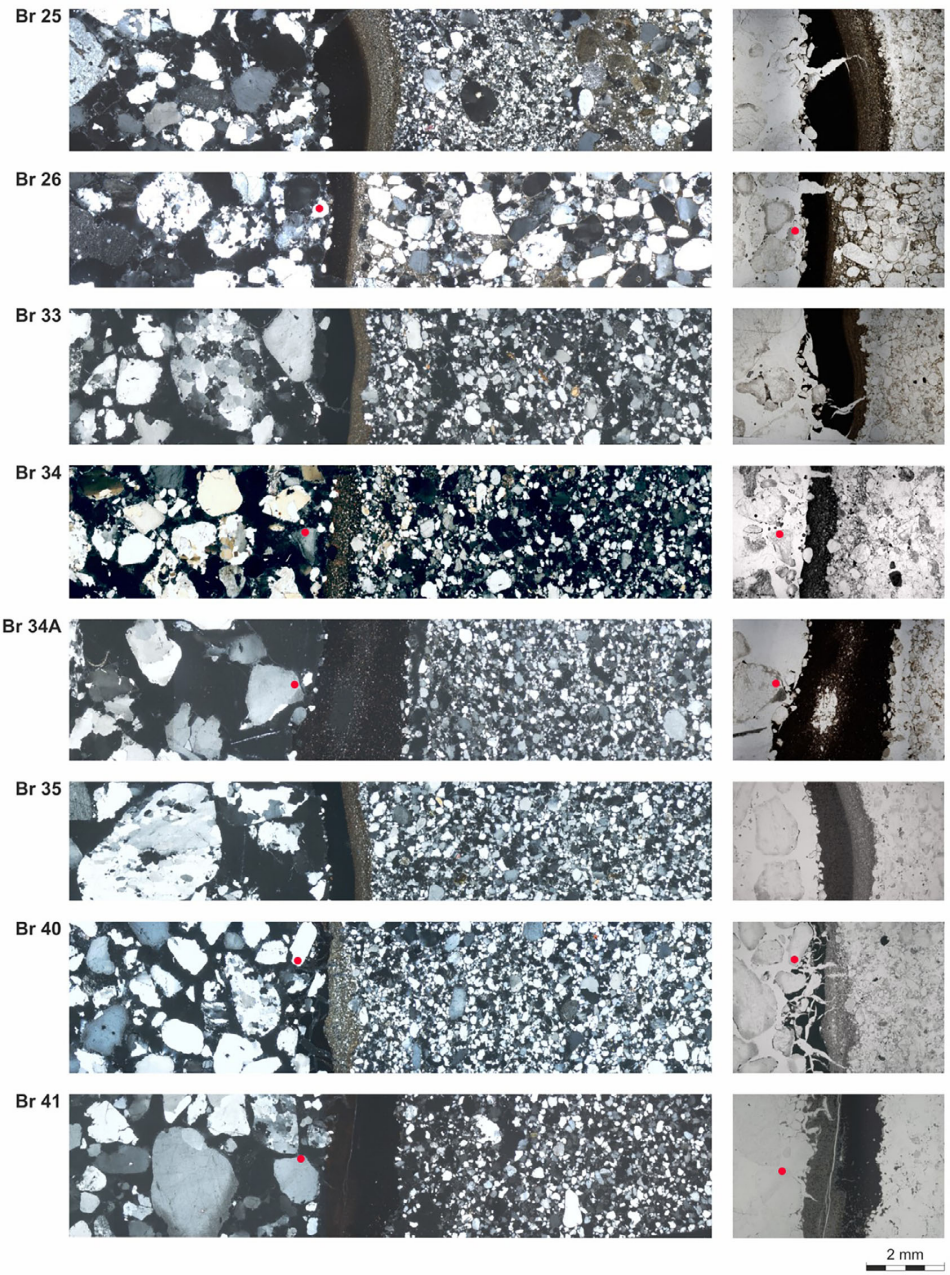
Microscopic thin section view of the borehole wall of the Reichwalde wells (composite images from several photographs). Left column: polarized light, right column: detail view under unpolarized light. In all images, the gravel pack is to the left, the skin layer roughly in the middle, and the aquifer material to the right. The red dots indicate features visible in both the polarized and unpolarized images (image pairs of Br 25, 33, and 35 do not overlap).

The thin sections of the borehole walls of all wells show some similarities but also some variability, both in thickness and typology (Figure 5). In most cases, the skin layers show a distinct layering, comprised of a coarser, more silicate‐dominated outer layer and one more homogeneous and compact inner layer. The latter probably inherits its brownish color from incorporated organic material, most probably stemming from the lignite seams. The absence of a distinct brown layer in the wellbore skin of Br 34, which did not reach the main seam, confirms this assumption. Since the main lignite seams are perforated late in the drilling process, due to its depth, it makes sense that the organic rich layer is deposited later, at the inner side of the borehole wall. A similar layering had already been described in Houben et al. (2024). In some cases, both layers are of roughly equal thickness (e.g., Br 25, Br 35); in some cases, the organic layer is thicker (Br 26, Br 33), and in some cases, the silicate layer is thicker (Br. 24, Br 40). In two cases, however, the order seems to be reversed (Br 34, Br 41). Here, the outer layer is more fine‐grained and brownish. It could be speculated that the upper lignite may play a role here or that no significant silicate skin layer formed before reaching the main seam. All skin layers are present directly at the borehole wall and are thus of the “surface cake” type after McDowell‐Boyer et al. (1986). No significant infiltration of particles into the adjacent formation (deep bed filtration) was evident. Deposits of iron oxide incrustations in the gravel pack were not observed.

**Figure 6:**
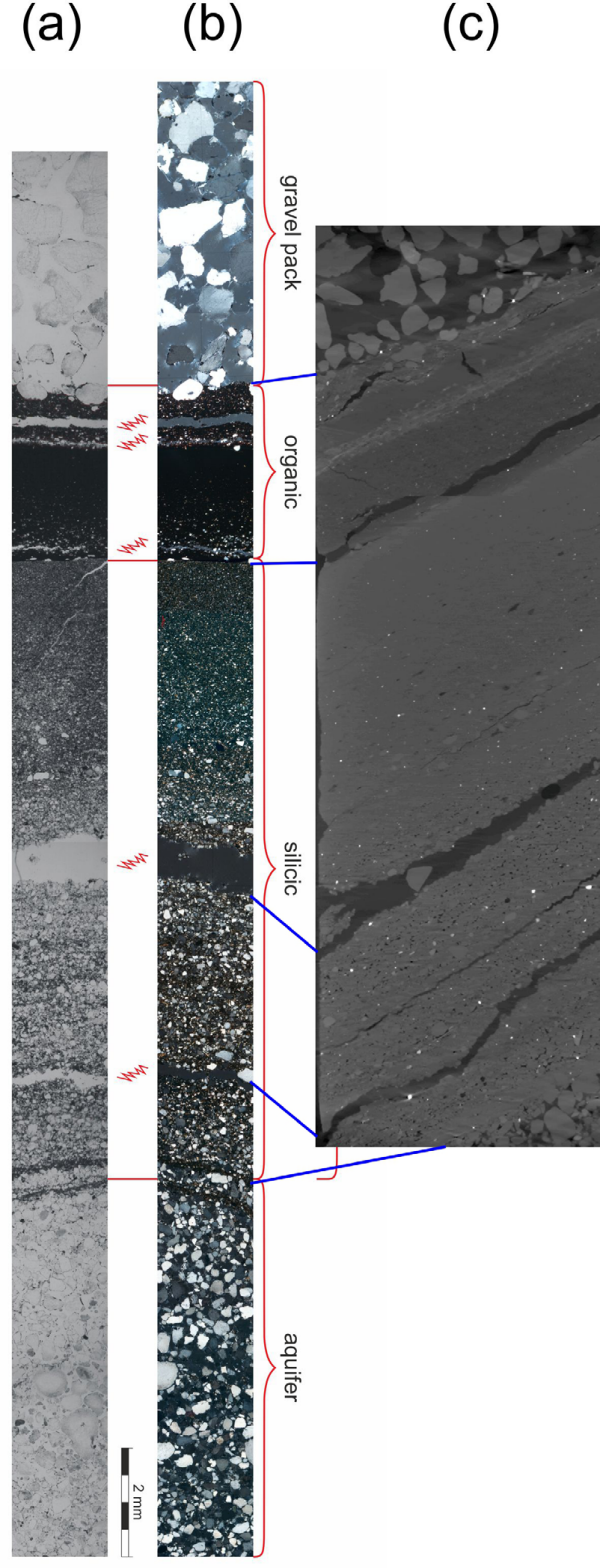
(a) microscopic thin section view of the borehole wall of Br 24 (composite from several photographs): left: unpolarized light, right: same, polarized light. The red wiggly lines indicate the presence of cracks, probably artifacts of sample preparation. (b) computed tomography image of a sample from Br 24 (side view). Blue lines connect features visible in both the thin section and the CT image. See Supporting Information for animation of CT images.

The unusually thick wellbore skin of Br 24 is displayed separately in Figure 6. The skin layer has a total thickness of 13 mm here. The maximum thickness observed in the field was around 25 mm. The inner, organic‐rich layer has a thickness of around 2 mm, while the remainder is mostly made up of silicate material, which shows a very distinct gradation. Both the microscopic section and the CT image agree that the material deposited first at the borehole wall is relatively coarse, while the material deposited later (toward the gravel pack) becomes successively more fine‐grained. This could be an indication of the succession of self‐sealing of the borehole wall. Initially, the borehole wall was very permeable, inducing high exfiltration velocities, carrying and depositing relatively coarse material. With this material deposited, the borehole wall permeability decreased and the exfiltration flow slowed down, both in rate and velocity, which in turn allowed only the transport and deposition of finer grains. As a final step, the very fine‐grained organic to clayey suspended material was deposited. Its high content of organic carbon suggests a source from the main lignite seam almost at the bottom of the borehole, which is reached at the end of the drilling process.

The Supporting Information contains animations made from image slices obtained by computed tomography (resolution = image distance: 7 μm) for wells Br 24, Br 26, and Br 33. For each wells, two view directions are available. The first (Videos S1a, S2a, and S3a) is perpendicular to the skin layer and thus has a sideway perspective (*xy* direction). The second follows a flow path of groundwater toward a well, going from the aquifer through the skin layer and then finally the gravel pack (*z* direction). Very bright particles, which sometimes pop up in the aquifer sediment and the skin layer, could be heavy minerals incorporated from the aquifer sediments but also (framboidal) pyrite crystals that may have formed in the sediment. The CT images nicely trace the layering of the skin layers and the porosity distribution, with higher porosities in the outer layer.

**Figure 7:**
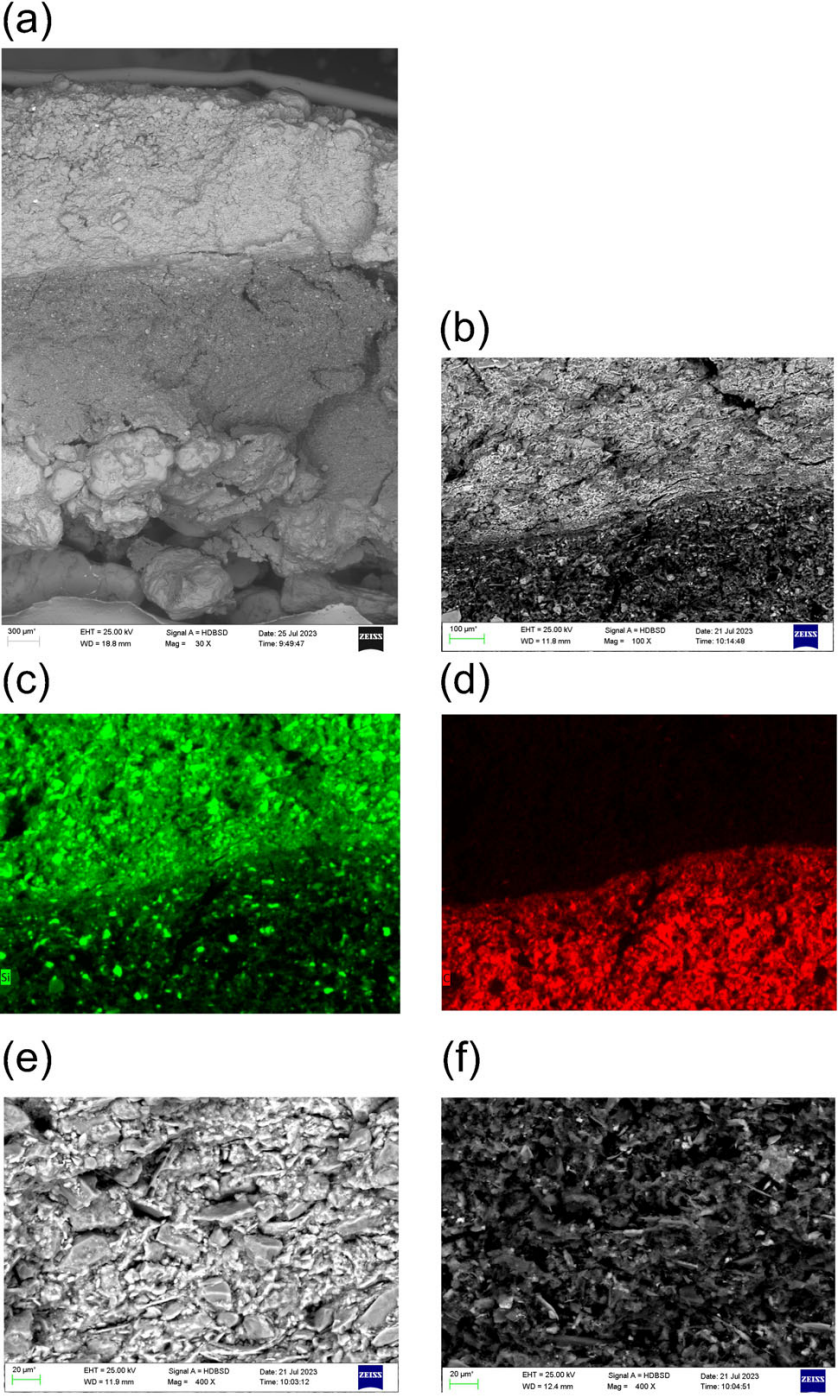
Scanning electron microscope images of the borehole wall of well Br 33: (a) total view: gravel pack (coarse, bottom) followed by light and dark skin layers. Composite image from three pictures, (b) detail view of the transition zone between light (above) and dark (below) skin layers, (c) element map for silicon (green) of the transition zone between light and dark skin layers (same display window as in Figure 7b), (d) element map for carbon (red) of the transition zone between light and dark skin layers (same display window as in Figure 7b), (e) detail view of light skin layer, (f) detail view of dark skin layer.

For the electron microscopy studies, only the results for well Br 33 are shown as an example (Figure 7). The skin layer again shows a twofold zonation, with an outer, light and an inner, dark layer (Figure 7a and 7b). Element mapping for silicon and carbon confirms the assumptions from the thin sections: the outer layer is mainly composed of silicate material (and almost devoid of carbon), while the inner layer is dominated by (organic) carbon, with some interspersed silicate grains (Figure 7c and 7d). Both layers show a parallel alignment of elongated and platy particles with the borehole wall.

### Granulometric and Hydraulic Properties

The grain size analyses show that the granulometry of the aquifer sediments for most wells is quite uniform, resulting in average hydraulic conductivities of around 5.65 × 10^−5^ m/s, calculated after the Beyer method (Table 2). Only Br 24 shows a distinctly coarser and better sorted sediment, resulting in hydraulic conductivities that are higher by up to an order of magnitude.

**Table 2:** Parameters for the Aquifer Material from Grain Size Analysis (C_U_ = Coefficient of Uniformity)

			Calculated Hydraulic Conductivity K (at 10 °C) (m/s)		
Code	Median Grain Size (μm)	C_U_ (= d_60_/d_10_)	BEYER	SEELHEIM	BIALAS
Br 24	320	2.11	3.00 × 10^−4^	3.66 × 10^−4^	1.02 × 10^−4^
Br 25	^a^	^a^	^a^	^a^	^a^
Br 26	^a^	^a^	^a^	^a^	^a^
Br 33	184	2.86	5.65 × 10^−5^	1.21 × 10^−4^	2.29 × 10^−5^
Br 34	199	3.17	4.97 × 10^−5^	1.42 × 10^−4^	2.12 × 10^−5^
Br 34A	180	3.41	3.60 × 10^−5^	1.16 × 10^−4^	1.73 × 10^−5^
Br 35	217	3.06	6.04 × 10^−5^	1.68 × 10^−4^	2.83 × 10^−5^
Br 40	201	2.91	5.91 × 10^−5^	1.45 × 10^−4^	2.59 × 10^−5^
Br 41	193	2.62	8.27 × 10^−5^	1.33 × 10^−4^	2.57 × 10^−5^

^a^ Could not be measured/calculated due to a higher proportion of the fraction <63 μm.

With hydraulic conductivities far beyond the measuring range of the permeameter, values for the conductivity of the gravel pack material were obtained from grain size analyses (not shown). Similar to the findings in Houben et al. (2024), they were found to uniformly group around K_gp_ = 2.5 × 10^−3^ m/s, a value that was then used for all further calculations.

The hydraulic conductivities of the aquifer material measured with the constant head permeameter and those obtained from granulometry are similar, although the former does not reflect the exceptionally high conductivity of the sediment of Br 24 well (Tables 2, 3). The values are well in range with previously measured results (Houben et al. 2016, 2024).

**Table 3:** Results of Constant Head Permeameter Tests: Measured Hydraulic Conductivities K for Aquifer and Total Sample and Calculated Conductivities for the Wellbore Skin Layer, Using Equation (1) from Houben et al. (2016).

		Total Sample (m/s) at 10 °C		Wellbore Skin		
Aquifer	Sample 1	Sample 2	Sample 1	Sample 2	Thickness^a^ (mm)
Br 24	5.03 × 10^−4^	6.95 × 10^−5^	5.10 × 10^−5^	7.55 × 10^−6^	5.42 × 10^−6^	5.0
Br 25	4.74 × 10^−5^	1.55 × 10^−3^	—	nd	—	0.8
Br 26	5.69 × 10^−5^	1.25 × 10^−4^	—	5.01 × 10^−6^	—	2.0
Br 33	4.23 × 10^−4^	1.66 × 10^−5^	—	3.05 × 10^−7^	—	0.9
Br 34	1.12 × 10^−4^	5.82 × 10^−5^	—	8.00 × 10^−7^	—	0.5
Br 34A	2.26 × 10^−4^	1.44 × 10^−5^	—	6.08 × 10^−7^	—	2.0
Br 35	9.71 × 10^−5^	2.85 × 10^−4^	—	nd	—	1.2
Br 40	2.39 × 10^−4^	5.15 × 10^−4^	7.52 × 10^−4^	nd	—	1.1
Br 41	6.83 × 10^−4^	1.38 × 10^−5^	—	5.60 × 10^−7^	—	2.0

Note: nd = could not be calculated.

^a^ Values measured on skin material taken from core (may be different from values recorded in optical analysis, due to natural variations).

### Mineral Composition

The quantitative mineralogical analysis of the wellbore skin underlines the differences between the individual samples. Some are clearly dominated by quartz and feldspar (Br 25, 26, 34), while others show very pronounced clay mineral contents (Br 33, 34A, 35, 41). For the thick skin of well Br 24, two samples were taken, one from the silicate‐dominated outer layer (Br 24‐1) and one from the organic‐rich inner layer (Br 24‐2). It is clear that the former contains much more quartz and feldspar, while the latter is strongly enriched in clay minerals, especially kaolinite, and organic material. The content of swellable clay phases was negligible for all samples (not shown).

### Comparison of Br 34 and Br 34A

The comparison of the wellbore skins of Br 34 and Br 34A allows interesting insights into the influence of the total drilling depth and the main lignite seam. Well Br 34 had to be abandoned at a depth of 60 m and did not penetrate the main seam. Its replacement well, Br 43A, drilled only 6 m away, thus limiting the effects of geological heterogeneity, reached the intended final depth and also fully penetrated the main lignite seam.

As a result, the skin layer of Br 34 is much thinner than that of Br 34A (Figure 5), indicating that a greater drilling depth allows for more mobilization and re‐deposition of particles from the penetrated formations. Furthermore, the skin layer of Br 34 comprises mostly quartz and feldspar (90 weight %), while the sample from Br 34A contains a far higher proportion of clay minerals (40 weight %; Table 4). The absence of an organic‐rich layer in Br 34 shows that the lignite seam also contributes to the skin formation. It should be noted that well Br 34, unlike Br 34A, never became operational and was immediately backfilled after its incomplete drilling. Influences of a later operation of Br 34A can thus not be ruled out as a cause of the observed differences.

**Table 4:** Quantitative Mineral Composition (K = Potassium) and Organic Carbon Content (C_org_) of Skin Material

Sample	Quartz (Weight %)	Plagioclase (Weight %)	K‐Feldspar (Weight %)	Muscovite (Weight %)	Kaolinite (Weight %)	Chlorite (Weight %)	Other (Weight %)	C_org_ (Weight %)
Br 24–1	56	9	9	11	10	1	2	0.97
Br 24–2	33	3	7	14	39	2	2	13.6
Br 25	68	4	10	8	9	1		1.71
Br 26	71	7	9	5	7	1		1.51
Br 33	50	6	9	11	22	1	1	10.7
Br 34	78	5	7	5	5			0.55
Br34A	44	7	8	14	26		1	9.56
Br 35	51	6	8	12	20	2	1	5.81
Br 40	61	5	8	10	15	1		2.70
Br 41	49	9	7	15	19	1		4.70

## Discussion

The results obtained so far provide several new insights into the interplay between particle‐providing and particle‐depositing layers and are thus helpful to understand and predict the formation of wellbore skin:

### Drilling Fluid Density and the Role of Particle‐Generating Layers

Although the drillholes, for which the drilling fluid densities were compared, are from similar geological formations, significant differences in the particle load of the drilling fluid and, to some degree, its grain size distribution are observed. It is interesting to notice that the geological differences between the two boreholes Br 5 and Br 14 are small. Well 14 apparently receives its much higher particle load from two distinct, but relatively thin source layers, a thin clay layer and a silt layer. In general, this shows that relatively small layers, which can be easily overlooked during drilling, can contribute the bulk of the particle load needed to deposit a significant wellbore skin layer. This effect of small‐scale geological heterogeneity, in turn, can explain why even neighboring, very similar wells may experience significantly different effects of wellbore skin. This, in turn, suggests that close and continuous monitoring of the drilling fluid parameters during drilling can help identify problematic zones. Especially sudden increases in fluid density should be considered a warning signal. Finally, these data might be used for future wellbore performance and stability predictions, for example, using artificial intelligence methods (e.g., Gomaa et al. 2019; Thabet et al. 2024; Xu et al. 2024; Davoodi et al. 2025).

A simple calculation based on the geometry of the borehole and the particle load shall show the effects: assuming a water column of 100 m during drilling and a diameter of 0.75 m, the borehole contained around 44,200 L of water (static volume). With an average particle load of 3 g/L for Br 5 and 10 g/L for Br 14 (taken from Figure 2), the drilling fluid contained 132.6 kg of particles for Br 5 and 442 kg for Br 14, respectively. This shows the much higher potential for wellbore skin deposition for Br 14. This calculation is static only; however, it neglects the continuous mobilization, recirculation, and deposition of skin material during the drilling process. The total particle masses involved must thus be higher. Assigning a typical bulk density of 1685 kg/m^3^ for packed sand, the mass of 442 kg would correspond to a volume of 0.260 m^3^. Considering the presence of less dense clay material and organic matter in the skin material, the actual volume would be higher. Distributing the volume of 0.260 m^3^ over the wetted surface area of the borehole of 235.6 m^2^ yields an average skin thickness of 1.1 mm, close to observed values.

### Wellbore Skin Variability and its Causes

The wellbore skins from the excavated dewatering wells studied here and in previous publications show a remarkable variability in typology, composition, hydraulic conductivity, and thickness. While some of the variations have been explained successfully (Houben et al. 2016, 2024), especially the variations in thickness have eluded a consistent theoretical approach. Houben et al. (2024) speculated that the thickness of wellbore skin layers increases with drilling depth, based on a rather small set of samples, though. However, the results from the Reichwalde wells suggest a different explanation. The study on the drilling fluids indicates that a significant skin layer can only form if non‐cohesive and poorly sorted layers are present, which supply the necessary load of particles. These layers can be rather thin and might be overlooked when applying only the rather coarse tools usually available on the drilling site (e.g., relying just on the drilling log). To at least monitor this issue, it is recommended to track the drilling fluid density over the course of the drilling. High densities indicate potential wellbore skin buildup, and sudden jumps in fluid density indicate the depth of the problematic layer. With very high loads, it could be useful to replace the drilling fluid with fresh water, especially before reaching the target aquifer.

Mobilized particles will be carried preferentially toward the most permeable parts of the borehole wall, where most of the exfiltration into the aquifer occurs, at least initially. The bulk of the suspended particles will be deposited there, slowly decreasing the borehole permeability, thus doing the job intended for drilling fluid additives, that is, decreasing drilling fluid losses. The continuous plugging of the borehole surface there leads to a decrease in fluid velocities, which in turn explains the internal gradation of the wellbore skin from coarse to fine grain sizes. The highest skin thicknesses should be expected in the (initially) most permeable layers. Well Br 24 is a good example for this: compared to the other Reichwalde wells, its median grain size and permeability are significantly higher (Table 2), the latter by an order of magnitude.

### Limits of Current Study

Despite our sample set probably being the largest of actual well skin samples worldwide, the number of samples and data derived from them is still relatively small, with all of them coming from a few sites in Germany, almost all from dewatering wells, which differ from water supply wells in some issues e.g. the absence of well development. Considering the natural heterogeneity in aquifer hydrogeology and the variety of drilling techniques available, it might be a bit too bold to generalize our results as representative just yet.

## Conclusions

The wellbore skin samples taken from nine very similar dewatering wells of the Reichwalde surface mine and the drilling fluid samples taken from wells from Nochten significantly expand our knowledge and understanding of the interplay between particle‐producing and particle‐absorbing layers in a borehole and the resulting variations in wellbore skin composition and properties:

1. The formation of a wellbore skin requires the presence of one or more layers which release particles. Non‐cohesive and poorly sorted material is the most likely candidate.
2. Not all perforated clay layers necessarily contribute to the formation of wellbore skin; cohesive clays may resist erosion.
3. Particle‐releasing layers can be thin and thus easily overlooked during drilling. It is recommended to monitor the density of the drilling fluid during the drilling process in order to assess the probability of skin formation and the potential thickness of particle‐producing layers.
4. Lignite seams contribute to the formation of wellbore skin. Due to the low density of the material, its contribution is more by volume and not by mass.
5. The thickest wellbore skin layers form at the (initially) most permeable layers, which absorb the highest rates of exfiltration. A flow meter inspection can be useful to identify such layers.
6. Wellbore skin layers show a gradation with coarser silicate particles deposited first at the borehole wall, followed by finer particles with decreasing exfiltration rates and finally very fine organic and clay material

These observations provide important new insights into the formation of wellbore skin, especially the role of the particle‐releasing and particle‐absorbing layers. This can help drillers and operators in building wells less affected by wellbore skin, for example, by thorough hydraulic well development, focusing on the most strongly affected zones. Continuously monitoring the drilling fluid density during the drilling process can help identify problematic layers, especially when sudden increases in density occur. Highly loaded drilling fluids should be freshened or replaced prior to entering the target aquifer.

## Authors' Note

The authors do not have any conflicts of interest or financial disclosures to report.

## Supporting information


**Video S1a.** Animation of computed tomography (CT) images of sample Br 24, sideway view (*xz* direction). Image width ca. 10 mm, resolution 7 μm.

**Video S1b.** Animation of CT images of sample Br 24, proceeding vertically (*z* direction, in direction of flow), showing the sequence of gravel pack, skin layer and aquifer. Image size ca. 10 mm × 7 mm, resolution 7 μm.

**Video S2a.** Animation of CT images of sample Br 26, sideway view (*xz* direction). Image width ca. 10 mm, resolution 7 μm.

**Video S2b.** Animation of CT images of sample Br 26, proceeding vertically (*z* direction, in direction of flow), showing the sequence of gravel pack, skin layer and aquifer. Image size ca. 10 mm × 8 mm, resolution 7 μm.

**Video S3a.** Animation of CT images of sample Br 41, sideway view (*xz* direction). Image width ca. 10 mm, resolution 7 μm.

**Video S3b.** Animation of CT images of sample Br 41, proceeding vertically (*z* direction, in direction of flow), showing the sequence of gravel pack, skin layer and aquifer. Image size ca. 7 mm × 8 mm, resolution 7 μm.

## Data Availability

Measurement data and protocols are available from the authors. The copyright for all images and photos lies with the authors.
